# Lifestyle, Working Conditions, and Quality of Life Among Dentists in Kazakhstan

**DOI:** 10.1155/ijod/4290036

**Published:** 2024-11-25

**Authors:** Azhar Syzdykova, Karlygash Toguzbayeva, Aiman Syzdykova, Danara Bekkazinova, Ainur B. Qumar, Akmaral Abikulova, Aigulsum Izekenova

**Affiliations:** ^1^Department of Public Health, Asfendiyarov Kazakh National Medical University, Almaty, Kazakhstan; ^2^Department of Therapeutic and Pediatric Dentistry, Kazakh-Russian Medical University, Almaty, Kazakhstan; ^3^Quality Control Department, OSMK LLP “INVIVO”, Almaty, Kazakhstan; ^4^Health Policy and Management Department, Asfendiyarov Kazakh National Medical University, Almaty, Kazakhstan; ^5^Epidemiology with HIV Courses Department, Asfendiyarov Kazakh National Medical University, Almaty, Kazakhstan

**Keywords:** dentists, Kazakhstan, lifestyle, quality of life, working conditions

## Abstract

**Background:** The study of working conditions and the quality of life (QoL) of medical workers has not lost its relevance worldwide. This highlights the need to understand the many aspects of life that may characterize physician performance and satisfaction with working conditions. This is extremely important for providing quality and safe medical care. This research aims to study dentists' lifestyles and working conditions in the Republic of Kazakhstan and the impact of these indicators on their QoL.

**Methods:** This article presents the results of a cross-sectional survey of a sociological questionnaire conducted with the participation of 254 dentists in Kazakhstan.

**Results:** Only a third of dentists follow a healthy lifestyle. In total, 39% of dentists currently smoke, and 24% drink alcohol. Dentists with more than 20 years of experience have a worse QoL and have the lowest scores in all domains compared to dentists with less experience. The lowest score was scored for mental health (mean = 51.81). On all scales, dentists who worked more than 40 h a week reported decreased QoL; the lowest score was in the “mental health” domain (mean = 52.80).

**Conclusion:** The QoL of dentists in Kazakhstan is negatively affected by their working hours and long working experience. Measures are necessary to improve their working conditions, work schedule planning, and mental health.

## 1. Introduction

Like other healthcare professionals, dentists' well-being is related to their quality of life (QoL) [1]. Many factors, such as lifestyle, area of specialization, income received from a dental practice, relationships with colleagues, social life, marital status, etc., affect the QoL of healthcare professionals [2–4].

Some studies show dentists are often exposed to occupational health hazards such as stress, high workload, and ergonomic and mental stress [5, 6].

The concept of QoL, defined by the World Health Organization (WHO) as “people's perception of their position in life in the context of culture and value systems,” encompasses various aspects, including physical health, psychological well-being, social relationships, personal beliefs, and the environment [3]. The influence of working conditions on health has been extensively researched over the last two decades [7–9]. The physical work environment of dentists is characterized by high exposure to awkward postures [6]. The interaction of poor working conditions may predict adverse health outcomes, including fatigue, anxiety, depression, and physical illness [10].

In 2022, about 570 public and 1165 private dental clinics will be operating in the country, with the total number of dentists in Kazakhstan being 4911 people [11]. Following the Labor Code of the Republic of Kazakhstan, the average period an employee performs his duties by the employer's acts should not exceed 40 h per week [12]. Work–life balance and well-being are one of the essential ways to maintain and provide a high level of patient care [13].

In a recent study, decreased performance in terms of loss of productivity was associated with poor sleep quality, high stress levels, and pain in multiple locations [14]. Stress, mental strain, and high workload are common problems, but little research has been done into these issues in the dental profession [15].

Lifestyle and QoL concepts are critical factors in developing strategies and programs to improve the well-being and living standards of the population [1]. Lifestyle and QoL measures are key health indicators and can influence healthcare providers' decisions regarding patients and public health policy [16]. Addressing these issues can improve people's QoL and a healthier societal environment.

This research aims to study dentists' lifestyles and working conditions in the Republic of Kazakhstan and the impact of these indicators on their QoL. The results may lead to improvements in the work environment of healthcare managers.

## 2. Methods

### 2.1. Design

A cross-sectional survey was conducted on 254 dentists working in dental clinics in Almaty. The study used a specially designed questionnaire and the methodological principles of a sociological survey. The subject of the study was dentists' lifestyles, working conditions, and QoL. The survey was conducted with respondents' informed consent in compliance with ethical standards.

Differences between such characteristics as age, work experience, working time, and factors related to a healthy lifestyle were assessed. In addition, the relationships between the occurrence of factors related to (1) a healthy lifestyle, (2) working time and how many places the doctor works, and (3) aspects of QoL were analyzed.

### 2.2. Participants

An invitation letter describing the study and its purpose was sent to the private dental medical centers' email. Then, a link to the questionnaire with explanations of the goals and objectives of the study was sent by phone numbers in WhatsApp Messenger to the center's staff. An online questionnaire was distributed using a snowball technique among dentist staff of private dental clinics in Almaty. The questionnaire was then filled out by respondents independently.

Almaty city has 937 dentists in dental clinics [11]. The nonprobability convenience sampling approach was used to select the respondents. The estimated minimum target sample size was half of the population—468, measured with a confidence interval of 95%, an acceptable margin of error of 5%, and an expected frequency of 50%. This elevated proportion enhances the sample's strength. Responses were received from 305 dentists working in 54 dental clinics. However, according to the inclusion criteria—dentists with at least 1 year of work experience, only 254 responses were selected.

All respondents were informed about the study, which included a description of the research and its purpose. The data collection period was between September 10, 2022 and January 1, 2023.

### 2.3. Instrument

This cross-sectional study used a prevalidated questionnaire for 4 weeks, from August 1 to September 1, 2022. The questionnaire was initially made in Kazakh and Russian.

Two instruments were used for obtaining the data: (1) a questionnaire of the authors' design and (2) the SF-36 questionnaire [17]. In total, the questionnaire included 56 questions for self-completion and grouped into sections: demographic information, working conditions, QoL, and aspects of lifestyle. The sociodemographic factors studied included age, education, and marital status.

The 12-item Health Literacy Questionnaire (HLQ) instrument was adapted to study healthy lifestyles. Five of them assess factors associated with a healthy lifestyle: tobacco use (e.g., I smoke regularly), sleep habits (e.g., I sleep between 7 and 8 h at least five times a week) regarding maintaining meal times (e.g., I eat breakfast, lunch, and snacks at the same time, at least five times a week), and maintaining a balanced diet (e.g., “I eat five servings of fruits and vegetables every day” at least five times a week). Responses were collected on a Likert scale with scores ranging from 1 (strongly disagree) to 5 (strongly agree).

The quality-of-life part of the questionnaire consisted of the universal SF-36 questionnaire, validated, and used in many national and international studies to assess QoL [18]. The answer options were on a Likert scale ranging from 1 to 100 points, and a better QoL earned a higher score. The SF-36 questionnaire consists of 36 points, divided into eight domains: general health perception (five items), physical functioning (10 items), social functioning (two items), role limitations due to physical problems (four items), role limitations due to emotional problems (three items), mental health (five items), vitality (four items), pain intensity (two items), and a single health transition item. Raw domain scores are transformed to a scale of 0–100, with high scores indicating better health status.

Before the survey, four academicians validated the draft questionnaire before ensure its relevance, adaptability, and consistency in our setting, as well as to define its face and content validity. Twelve people participated in its pilot program to provide comments, suggestions, and feedback on its intelligibility, transparency, ease of use, and question clarity. The final analysis did not incorporate the results from the pilot study. Still, the input was examined, leading, double-barreled, and confusing questions were fixed, and a finalized version of the questionnaire was created.

### 2.4. Statistical Analysis

Data were processed using Statistical Package for the Social Sciences (SPSS) version 21.0 (SPSS Inc., International Business Machines [IBM]). To present continuous data, average values, and standard deviations (SDs) were used, with the significance of differences in indicators assessed using the Student's *t*-test.

To prepare and examine the data, statistical analyses included a one-way analysis of variance (ANOVA), linear correlation analysis, and a linear regression model. The regression model was employed to identify the factors significantly influencing health behavior levels.

A nonparametric statistical test, ANOVA, and the Bonferroni test were used post hoc with Kruskal–Wallis. Effect sizes were calculated for all comparisons. Pearson's correlation coefficient was used for scale variables, and Spearman's association with ordinal variables was used to relate the quality-of-life scores to other variables. The accepted significance level was *p* < 0.05.

### 2.5. Procedure

#### 2.5.1. Ethical Approval

The study was approved by the Local Ethics Committee (IRB00011496, protocol No. 13(119) from September 29, 2021) of Asfendiyarov Kazakh National Medical University, Almaty, Kazakhstan. At the beginning of the questionnaire, respondents were required to sign a participant informed consent form, so a fully completed survey confirmed their consent to complete the questionnaire. Respondents were guaranteed confidentiality and anonymity of personal data.

## 3. Results

The mean age of the 254 participants (46.4% females) was 39.15 ± 10.22 years (ranging from 25 to 58 years). The distribution of doctors by age was as follows: 25–34 years (34.6%), 35–44 years (35.0%), 45–54 years (21.3%), and over 55 years (9.1%). The background characteristics of the participants are shown in Table 1.

Average actual working hours per week ranged from 30 to 42 h. In total, 175 (68.9%) respondents worked in two or more medical organizations.  Table 2 presents the participants' engagement in health-risk behavior.

Analysis of data from a lifestyle study showed that 34.6% (*n* = 88) of respondents slept quality sleep (sleep enough hours [7–8 h] per day), 5–6 h—42.6% (*n* = 108), and less than 5 h—22.8% (*n* = 58) of doctors.

The study revealed that only 29.5% (*n* = 75) of dentists ate regularly with timely meals (three times a day), 48.4% (*n* = 123) ate balanced and regularly but only 1–2 times a day, and 22.1% (*n* = 56) ate unbalanced and irregularly.

Only 28.3% of participants (*n* = 72) exercised regularly, and 30.7% (*n* = 78) did not engage in physical activity at all.

Smoking, drinking alcohol, overeating, and a sedentary lifestyle play a significant role in poor health, being leading risk factors for many diseases. More than half of the respondents—54.7% (*n* = 139) smoked in the past, and 24.0% (*n* = 61) continued to smoke at the current time.

The majority of a third of respondents, 39% (*n* = 99), consume alcohol regularly. Only 26.4% of respondents (*n* = 67) never drank alcohol.

A study of QoL depending on doctors' working hours showed that general health, role-physical and social functioning, and vitality were reduced among doctors who worked more than 40 h a week (Table 3). The number of hours worked per week affects all indicators of QoL, general health, and mental health. The indicators of the “General health perception,” “role-emotional functioning,” “pain intensity,” “vitality,” and “mental health” scales decrease.

Doctors who worked more than 40 h per week scored the lowest on mental health (mean = 52.80), followed by social functioning (mean = 58.42). The highest score in this group was for pain intensity (mean = 70.56). Across the subscales, participants who worked up to 40 h per week scored the highest on role-physical functioning (mean = 80.71) and the lowest on role-emotional functioning (mean = 62.11).

Dentists with more than 20 years of experience scored the lowest in all domains compared to doctors with less experience (Table 4). The lowest score was mental health (mean = 51.81), followed by vitality (mean = 54.24). The highest scores were in the group of doctors with up to 5 years of experience. For the domain “general health perception,” the mean was 89.41, followed by “social functioning,” with a mean of 85.21. The lowest in this group was in the viability domain (mean = 71.30).

## 4. Discussion

The study aimed to investigate the lifestyle and working conditions of dentists in Kazakhstan and examine the impact of these factors on their QoL. The findings revealed several important insights. The results show that work duration affects dentists' QoL and functional abilities.

First, only a third of dentists in Kazakhstan reported following a healthy lifestyle. This indicates that a significant number of dentists may engage in behaviors that can negatively affect their overall well-being and QoL. Our study confirms the study of the lifestyle of orthopedic dentists, where most doctors, knowing the risk factors for the development of chronic pathology, did not carry out many elements of health-saving behavior [19]. With increasing work experience, indicators of physical functioning, role-emotional functioning, pain intensity, and mental health among respondents decreased.

Findings on smoking and drinking alcohol among third parties of dentists highlight the need for interventions and support systems to promote healthier lifestyles among dentists in Kazakhstan.

Furthermore, the study found that dentists with extended work experience had a worse QoL than those with less experience. This suggests that long-term exposure to the dental profession may have a detrimental effect on dentists' well-being. Healthcare organizations and policymakers should address the specific challenges faced by experienced dentists and implement measures to improve their QoL.

Additionally, the study found that dentists who worked more than 40 h per week had a decreased QoL across all domains, with the lowest scores observed in the mental health domain. This highlights the importance of work schedule planning and ensuring dentists have a healthy work–life balance. Implementing strategies to reduce excessive working hours and providing support for mental health can improve dentists' overall QoL.

All these results need to be interpreted with several analytical components in mind. First, dentistry in Kazakhstan is an entirely private practice. Dentists' behavior directly impacts their well-being, as they are often too busy with work and daily issues to prioritize health-improving procedures. This can lead to emotional burnout syndrome and the worsening of chronic health conditions. Lifestyle elements play a significant role in the development of these issues.

Qualitative findings showed that QoL is linked to the level of monthly income and consideration of work compensation [20]. Specialization in the job market is a transient profession with fewer job benefits compared to permanent contracts, but these work conditions are offset by the flexibility to balance work and personal life [21].

The QoL of dentists may play a significant role in patient care delivery and communication, affecting patient satisfaction with their treatment. Previous studies among dentists [16] have highlighted several factors positively associated with job satisfaction, including work environment, marital status [3], practice location, years of experience, and income. Factors such as relationships with patients and years in practice have also been found to impact job satisfaction [22].

Stress and job satisfaction have a complex relationship, and stress may be an essential feature of a dentist's job [3, 10]. Because burnout affects all aspects of life, including family problems, emotional distress, and substance abuse problems, it has a devastating impact on patients, leading to medical errors and decreased adherence to medical advice [12–14]. Although several studies have addressed the job satisfaction of dentists, the literature on QoL among dentists in general is sparse [11, 13, 22].

This study brings to current knowledge a growing need for doctors to actively apply their knowledge in maintaining and strengthening their health due to the increased pace of life and the intensification of work in the modernization of healthcare. As highlighted in the study, interventions and improvements in the working conditions of dentists in Kazakhstan are crucial.

## 5. Limitations of the Study

This study has several limitations that need to be noted. First, a cross-sectional design means that the data were examined at one point and only once. This design does not allow observing changes in working conditions, lifestyle, and QoL of dentists over a long period. This precludes the establishment of any convincing cause-and-effect relationships between lifestyle, working conditions, and QoL. Second, there may have been some physician selection bias and the sample was relatively small. Third, self-report bias may have occurred in online and self-administered questionnaires. Respondents may have desired to hide their lifestyle; thus, it is possible that what clinicians considered acceptable health behaviors may have been overrepresented in the results.

However, we used a tool that has been validated and widely used in research.

## 6. Conclusions

We observed the worst QoL among dentists with more than 20 years of experience and working more than 40 h per week.

Dentists rarely follow recommendations for optimizing lifestyle; only a third (29.5%) of dentists follow a diet and eat balanced foods. In total, 28.3% of dentists engage in physical activity regularly. In total, 39% of dentists currently smoke and 24% drink alcohol.

The QoL of dentists in Kazakhstan is negatively affected by their workload and working hours. In total, 68.9% of respondents worked in several organizations, and 24.41% worked more than 40 h a week.

Studying dentists' lifestyles and working conditions is necessary to ensure the quality of their lives and professional activities. Measures must be taken to improve their working conditions, work schedule planning, and other aspects to improve their QoL [5, 6].

## Figures and Tables

**Table 1:** Participants' demographic characteristics (*N* = 254).

Characteristics	Frequency (*n*)	Percentage
Age (years)		
25–34	88	34.6
35–44	89	35.0
45–54	54	21.3
More than 55	23	9.1
Gender		
Male	136	53.5
Female	118	46.5
Marital status		
Single/not married	61	24.0
Married/living together	139	54.7
Divorced	54	21.3
Work experience in specialty		
5 years or less	54	21.3
6–10 years	62	24.4
11–15 years	67	26.4
16–20 years old	38	14.9
Over 20 years	33	13.0
Place of residence		
City	158	62.2
Village	96	37.8
Working hours (per week)		
11–20	5	1.97
21–30	45	17.72
31–40	142	55.91
41–50	62	24.41

**Table 2:** Participants' health-risk behaviors (*N* = 254).

Health-risk behaviors	Frequency (*n*)	Percentage
Rest habits		
I sleep between 7 and 8 h at least five times a week	88	34,6
5–6 h	108	42,6
Less than 5 h	58	22,8
Nutrition		
Balanced diet regularly (three times a day)	75	29.5
Balanced diet regularly (1–2 times a day)	123	48.4
Unbalanced and irregular diet	56	22.1
Physical activity		
Regularly (at least 150 min of moderate-to-vigorous intensity physical activity per week)	72	28.3
Irregular (less than 150 min of moderate-to-low-intensity physical activity per week)	104	41
Does not engage in physical activity	78	30.7
Cigarette use		
Never	54	21.3
Past but not current	139	54.7
Current	61	24
Alcohol use		
Never	67	26.4
Past but not current	88	34.6
Current	99	39

**Table 3:** Median SF-36 total work time scores.

SF-36 scale	<40 h per week		>40 h per week		
	MD	SD	MD	SD	*p*
General health perception	80.52	7.16	68.81	21.90	0.032
Physical functioning	72.13	31.10	61.12	30.42	0.502
Role limitations due to physical problems	80.71	14.12	69.20	19.01	0.581
Role limitations due to emotional problems	62.11	18.31	59.04	17.65	0.018
Social functioning	64.24	19.63	58.42	19.03	0.102
Pain Intensity	73.38	20.54	70.56	15.11	0.035
Vitality	64.72	31.16	59.10	30.12	0.014
Mental health	62.98	17.18	52.80	14.65	0.031

Abbreviations: MD, mean deviation; SD, standard deviation.

**Table 4:** Median SF-36 total scores by years of work experience.

Work experience	5 years or less		6–10 years		11–15 years		16–20 years		Over 20 years		
SF-36 score	MD	SD	MD	SD	MD	SD	MD	SD	MD	SD	*p*
General health perception	89.41	7.88	82.11	10.76	79.72	14.91	68.81	20.41	66.17	18.02	0.003
Physical functioning	81.74	23.65	78.04	31.75	75.00	36.12	60.87	31.00	60.11	18.90	0.025
Role limitations due to physical problems	80.04	12.54	75.08	18.30	76.16	20.41	62.16	22.84	60.12	31.01	0.015
Role limitations due to emotional problems	76.32	18.90	79.21	18.04	76.33	18.25	60.31	18.28	60.31	18.28	0.018
Social functioning	85.21	21.08	78.82	15.13	67.75	22.12	60.78	14.54	58.27	15.45	0.002
Pain intensity	78.21	25.33	78.40	21.13	74.12	21.71	63.24	20.31	60.18	21.30	0.011
Vitality	71.30	32.27	71.23	32.21	69.33	31.01	55.13	31.33	54.24	22.38	0.201
Mental health	76.34	18.17	75.40	17.50	69.71	18.01	57.86	18.82	51.81	19.12	0.018

Abbreviations: MD, mean deviation; SD, standard deviation.

## Data Availability

The datasets analyzed during the current study are available from the first author (Azhar Syzdykova) upon request.
